# Editorial: The Evolving Landscape of Stereotactic Body Radiation Therapy for the Management of Prostate Cancer

**DOI:** 10.3389/fonc.2020.627686

**Published:** 2020-12-15

**Authors:** Seth R. Blacksburg, Donald B. Fuller, Jonathan A. Haas

**Affiliations:** ^1^ Department of Radiation Oncology, Perlmutter Cancer Center at NYU Long Island Hospital, Mineola, NY, United States; ^2^ Department of Radiation Oncology, Genesis Healthcare Partners, San Diego, CA, United States

**Keywords:** prostate, radiotherapy, stereotactic, Stereotactic Body Radiation Therapy, prostate cancer

Over the last decade, an increasing number of publications have demonstrated the feasibility, safety, and efficacy in utilizing a condensed schedule of radiation to manage localized prostate cancer (1–4). Hypofractionation refers to delivering modestly larger doses than is prescribed with conventional regimens, while ultrahypofractionation refers to delivering an even larger dose with correspondingly fewer fractions. Stereotactic Body Radiation Therapy (SBRT) is the technique utilized to deliver ultrahypofractionation, and has become a standard regimen employed in the treatment of men with low and intermediate risk prostate cancer. This Research Topic highlights the evolving landscape of Stereotactic Body Radiation Therapy for the management of localized and advanced prostate cancer, addressing current data regarding clinicopathologic and dosimetric optimization with novel perspectives.

Numerous prospective phase I/II studies have shown favorable biochemical outcomes with the use of prostate SBRT (5–16). A study comprised of pooled single and multi-institutional trials from a cohort of 2142 patients demonstrated excellent 7-year biochemical outcomes for men with low, favorable intermediate, and unfavorable intermediate risk disease (17). A meta-analysis composed of over 6,000 patients highlighted the prospective evidence supporting the use of SBRT, concluding, “SBRT has sufficient evidence to be supported as a standard treatment option for localized prostate cancer while ongoing trials assess its potential superiority (18)”. Based on data from the NCDB and SEER database, there has been a contemporaneous increase in the use of SBRT for managing localized prostate cancer (19–22).

While most of the initial publications involving the use of prostate SBRT centered on the treatment of men with low and intermediate risk disease, patients with unfavorable intermediate or high risk disease were often times also included. The applicability of SBRT to men with unfavorable intermediate and high risk disease continues to evolve. An increasing number of series have characterized promising biochemical outcomes for this specific cohort (17, 23–26). Reflecting these emerging data, the National Comprehensive Cancer Network (NCCN) begrudgingly supports the use of SBRT for men with unfavorable intermediate or high risk disease “…if delivering longer courses of EBRT would present a medical or social hardship (27)”. Most recently, the Scandinavian HYPO-RT-PC trial randomized 1200 men with intermediate-risk or high-risk prostate cancer to conventional vs. stereotactic radiotherapy regimens. Eleven percent of the cohort harbored high risk disease. At a median follow-up of 5 years, there was parity in biochemical and late grade 2+ urinary or bowel outcomes (28). Ricco et al. review the literature pertaining to the use of prostate SBRT for men with intermediate and high risk disease and independently validate excellent 7-year biochemical control rates similar to that seen in the HYPO-RT-PC trial. Their findings and summary of data to date further advance the notion that patients with advanced localized disease can benefit from an SBRT approach.

Several series have reported a longer time to PSA nadir with a greater absolutely magnitude in biochemical response with SBRT when compared with conventional fractionation. These findings are consistent with higher biological effective dosing, which is also found in brachytherapy. (29–31) There is emerging data which suggests an improvement in biochemical outcomes with SBRT dose escalation (10, 32, 33). Whereas most series have accomplished this with homogeneous dosing on robotic or gantry-based platforms, heterogeneous-dosing methods employing a virtual HDR-brachytherapy approach with ablative dosing, has been described, with favorable biochemical and quality of life results (34). Fuller et al. reviews this technique with two different dosing schemas. With both doses, favorable biochemical outcomes were obtained, with modest rates of low toxicity. Given a measurable differential in the absolute magnitude of PSA ablation and quality of life outcomes between dose arms, the authors suggest the ability to utilize age and baseline clinicopathologic features to select between these doses when utilizing this approach.

With the increased utilization of prostate SBRT, there were concerns regarding its use portending for a potential decrease in quality of life. One series explored an increase in genitourinary toxicity through querying Medicare claims data (35). Another analysis had similar findings using data gleaned from the SEER database (20). Since, several series have reported highly favorable patient and physician-reported quality of life outcomes for patients treated with these abbreviated regimens (9, 17, 36, 37). Favorable quality of life outcomes has been further validated by initial data reported from a phase 3 trial comparing 38 Gy in 5 fractions with 79.2 Gy in 44 fractions (38). The PACE-B study randomized men with localized prostate cancer to treatment with conventional, hypofractionated, or SBRT regimens and had a similar finding of quality of life parity between treatment arms (39). Aghdam et al. report further contribute to our understanding of this subject, concentrating on an older patient cohort receiving prostate SBRT. Their findings illuminate patient characterization of disease, and treatment burden, with a minority reporting high long-term burden for either.

The role of pre-treatment clinical factors for predicting long-term quality of life after prostate SBRT continues to mature. The probability of developing benign prostatic hypertrophy and being diagnosed with prostate cancer independently increase over time. For patients that fail alpha-1 adrenergic receptor antagonists and/or 5-alpha reductase inhibitor medication, Transurethral Resection of the Prostate (TURP) or a derived variant of this procedure, is often prescribed. The role of pre-treatment TURP portending for genitourinary toxicity post definitive LDR brachytherapy or IMRT has been dismissed in select series (40–43). The data regarding pre-existing TURP after SBRT is currently emerging, with mixed findings that appear dependent, in part, on dose (44, 45). Pepin et al. clarify this question by reporting on long-term outcomes for 47 patients treated with modest SBRT dosing for whom previous TURP predicted for transient hematuria with comparable long-term toxicity to conventionally fractionated regimens.

Several series have characterized intrafraction motion during the course of prostate radiotherapy (46–50). Most prostate SBRT regimens employ anisotropic PTV margins between 3-5mm’s. Dose escalated HDR-like SBRT approaches use smaller margins of 0-2 mm, expanded to 5 mm adjacent to actual biopsy or MRI-demonstrated peri-capsular disease only, to minimize the risk of adjacent tissue injury (34). Levin-Epstein et al. characterize inter and intra-fractional prostate motion in 205 patients enrolled on two prospective studies of prostate SBRT. Their findings largely validate current stereotactic approaches which focus on accuracy and precision. Interestingly, inter and intra-fractional prostate displacement did not predict for grade 3+ toxicity.

Despite an increase in the number of series published on prostate SBRT and the corresponding acceleration in its adoption, universally recognized dosimetric predictors of toxicity after prostate SBRT remain elusive, with institutional and prospective trial planning objectives frequently based on BED calculations and legacy dose constraints. Publications centered on dose-volume objectives and quality of life outcomes are in want, with differing endpoints and assessments found in select series (44, 51). Valle et al. apply a sophisticated machine learning technique to assess 910 dosiomic features in predicting grade 2+ genitourinary toxicity for 339 patients treated with prostate SBRT at an academic institution. Their findings validate the use of advanced modeling for toxicity prediction, and highlight the need to incorporate biologic and genomic data in future analyses.

Several series have suggested an improvement in outcomes for men with metastatic prostate cancer with treatment to the primary site, culminating in the recent findings published from the STAMPEDE trial (52–57). There is also emerging data addressing potential benefit of stereotactic radiation directed to sites of oligometastatic disease (58). Adorno Febles et al. provide us with a comprehensive view of the rationale, utilization, and evolving data regarding the use of prostate SBRT and elucidates the potential interplay with systemic therapies for managing advanced disease states. A detailed exploration of SBRT and tumor immunology is explored along with a review of ongoing SBRT trials combined with systemic therapeutics.

As a whole, the current Research Topic aggregates a collection of articles which provide a high-level review of the rationale and evolution of the use of SBRT for the treatment of prostate cancer. It investigates outcomes involving distinct patient cohorts, techniques, and treatment planning objectives as monotherapy and in concert with systemic therapeutics. Ample data has suggested the ability of prostate SBRT to provide an efficacious substitute for costlier and more invasive and/or inconvenient regimens involving radiation therapy. As our understanding of optimal patient selection, techniques, prescriptions and dose objectives continue to develop, the potential for prostate SBRT to become a universally accepted *preferred* radiation therapeutic approach remains tantalizingly close.

## Author Contributions

The authors SB, DF, and JH have contributed equally to the editorial writing process. All authors contributed to the article and approved the submitted version.

## Conflict of Interest

The authors declare that the research was conducted in the absence of any commercial or financial relationships that could be construed as a potential conflict of interest.
